# 

**Published:** 1894-11-24

**Authors:** 


**Notices of Births, Deaths, and Marriages** are inserted in this column at a charge of 2s. 6d. for an announcement not exceeding four lines.

**Notice to Advertisers and Subscribers.**

All Advertisements, Orders for Copies of The Hospital, and Business Communications should be addressed to **The Publisher, NOT TO THE EDITOR,** The Hospital, 428, Strand, W.C.

The Cost of Subscription, post free, is as follows:—Per week, 2½d.; per quarter, 2s. 8d.; per half-year, 5s. 3d.; per annum, 10s. 6d. Foreign:—Per Annum, 12s. 8d.; payable, in all cases, in advance.

NOTICE TO CORRESPONDENTS.

All MS., Letters, Books for Review, and other matters intended for the Editor should be addressed The Editor, The Lodge, Porchester Square, London, W.

The Editor cannot undertake to return rejected MS., even when accompanied by stamped directed envelope.

**"Burdett's Hospital Annual," 1894.**

Fifth year of publication. 900 pp. Boards, gilt lettering. A most complete guide to British, Colonial, Indian, and American Hospitals, Dispensaries, Nursing and Convalescent Institutions, and Asylums. Price 5s. Published by The Scientific Press (Limited), 428, Strand, W.C.

**NOTICE.—The Index for Vol. XVI. (ending September 24th, 1894) is on sale at the Office of this Journal, Price 2½d., post free.**

### SCRAPS AND GLEANINGS.

A Hospital Sunday is proposed for the Cork district.

The statue of the late Dr. Marion Sims was unveiled at New York on November 13th.

The late Mr. T. Edwards Moss, M.P., has left £1,000 to the charities of Wildness, which is to be devoted to the nursing of the sick poor.

The largest sum yet collected through the Derby Saturday Fund, amounting to £1,229, has been handed over to the Royal Infirmary, less expenses.

An optometer for testing the sight has been presented to the West of England Eye Infirmary at Exeter by Messrs. Curry and Paxton. A legacy of £200 has also been bequeathed to the same institution.

It is proposed to extend the City Hospital for Infectious Diseases to the extent of 60 beds, besides improving the nurses' accommodation which is acknowledged as inadequate. The City Council desire to borrow £25,000 for the purpose. The movement is being strongly opposed by property holders.

The report of the Cork Street, Fever Hospital, Dublin, at the last monthly meeting of the Managing Committee, showed that half the present patients are small-pox cases, and that the diphtheretic ones are in excess of those in former years. Enteric fever has diminished, but scarlatina is rather prevalent. A case of small-pox, reported at the South Dublin Union, caused alarm to the public, as arrangements for disinfection are said to be defective.

### THE STUDENT OF MEDICINE.

### APPOINTMENTS.

T. H. Agnew, M.R.C.S., L.R.C.P.Edin., appointed House Surgeon to the Royal Infirmary, Liverpool. H. Armstrong, M.B., Ch.B.Vict., appointed House Physician to the Royal Infirmary, Liverpool. G. T. C. Barber, M.R.C.S.Eng., L.R.C.P.Lond., L.S.A., appointed Resident Medical Officer to the Birmingham Workhouse. A. Cohen, M.A., M.D., B.Ch. Dub., appointed Physician-in-Charge of Jewish Out-patients to the Metropolitan Hospital of London. Dr. C. Coles, appointed Medical Officer for the Fifth District of the Leicester Union. G. E. Coulson, M.R.C.S.Eng., L.R.C.P.Lond., appointed Assistant Medical Officer of the Infirmary of the St. George's Union. C. C. Easterbrook, M.A., M.B., C.M., appointed Third Assistant Physician to the Royal Asylum, Morningside, Edinburgh. W. d'E. Emery, B.Sc.Lond., M.R.C.S., L.R.C.P., appointed Obstetric and Ophthalmic House Surgeon to the Queen's Hospital, Birmingham. F. W. Fullerton, M.B., B.S.Durh., appointed Medical Officer for the Sculcoates District of the Sculcoates Union. T. H. Galbraith, M.B., C.M.Aberd., appointed Medical Officer of the Workhouse of the Wolverhampton Union. D. Gunn, F.R.C.S., appointed Assistant Surgeon to the Royal Westminster Ophthalmic Hospital. H. T. Hinton, M.B., C.M.Aberd., appointed Certifying Factory Surgeon for the Heytesbury District. J. Hutton, M.R.C.S.Eng., L.R.C.P.Lond., appointed Medical Officer for the High Cross District of the Tottenham Union. Dr. G. F. Johnston, appointed Registrar to the Hospital for Epilepsy, Regent's Park. H. C. Lamport, M.B., C.M., appointed Certifying Surgeon for Factories and Workshops for the Lancaster Division. G. A. Lang, M.B., C.M.Aberd., appointed Visiting Medical Officer to the Forbes Dispensary, Inverness. W. E. Livesey, M.B., Ch.B.Vict., M.R.C.S., L.R.C.P., L.S.A., appointed Lock Hospital and Ophthalmological Assistant to the Royal Infirmary, Liverpool. W. McClelland, M.B., Ch.B.Vict., appointed Gynæcological Assistant to the Royal Infirmary, Liverpool. N. M. MacLehose, M.B., C.M.Glasg., appointed Assistant Surgeon to the Central London Ophthalmic Hospital. H. Mason, M.B.Lond., M.R.C.S., L.R.C.P., appointed Medical Officer to the Leamington Provident Dispensary. E. S. Medcalf, L.R.C.P.Edin., M.R.C.S.Eng., reappointed Medical Officer of Health for Hove. J. P. Nixon, M.B., Ch.B.Vict., appointed House Surgeon to the Royal Infirmary, Liverpool. Mr. J. O'Connell, appointed Assistant Medical Officer to the Workhouse of the Parish of Toxteth Park. W. H. Plaister, M.R.C.S Eng., appointed Medical Officer for the Lower Cross Ward of the Edmonton Union. W. Robinson, M.D., M.S., F.R.C.S.Eng., appointed Honorary Physician to the Sunderland Infirmary. S. J. Ross, M.B., Ch.B.Vict., appointed House Physician to the Royal Infirmary, Liverpool. A. E. Shaw, M.R.C.S.Eng., appointed Medical Officer for the Raynham District of the Walsingham Union. F. H. Simpson, M.B., B.S.Durh., M.R.C.S., L.R.C.P., appointed House Physician to the Queen's Hospital, Birmingham. H. Sinigar, M.R.C.S., L.R.C.P., appointed House Surgeon to the Queen's Hospital, Birmingham. F. S. Toogood, M.D.Lond. D.P.H.Lond., appointed Medical Superintendent of the New Infirmary, and Principal Medical Officer of the Workhouse at Lewisham, S.E. F. J. de C. Veale, M.B., Ch.B.Vict., appointed House Surgeon to the Royal Infirmary, Liverpool. H. Watson, M.D.Aberd., appointed Medical Officer for the Fifth District of the Parish of Norwich. H. R. Wilson, M.R.C.S., L.R.C.P., appointed House Physician to the Royal Infirmary, Liverpool. Ewen J. Maclean, M.D, C.M.Edin., appointed Physician-Accoucheur to the St. Pancras and Northern Dispensary.

### VACANCIES.

The following vacancies are announced this week, the amount of salary in each case being given after the name of the institution:

### Resident Medical Officers.

Birmingham General Dispensary.—Resident Surgeon. Salary £150 per annum. Applications and testimonials to A. Forrest, Secretary, before December 12th.

General Hospital, Birmingham.—Assistant House Surgeon; must possess surgical qualification. Appointment for six months, but may be held by re-election for a further period of six months. Board, residence, and washing provided. No salary. Applications to the House Governor by December 1st.

Great Northern Central Hospital, Holloway Road, N.—Junior House Surgeon. Board, lodging, and laundry provided. Applications and testimonials to Lewis H. Glenton Kerr, Secretary, by November 26th.

New Hospital for Women, 144, Euston Road.—Female Resident Medical Officer; fully qualified. Applications to Margaret M. Bagster, Secretary, by November 28th.

North Staffordshire Infirmary and Eye Hospital, Hartshill, Stoke-upon-Trent.—House Surgeon. Salary £120 per annum, increasing £10 a year, at the discretion of the committee. Applications and testimonials to R. Hordley, Secretary, before November 27th.

Royal Westminster Ophthalmic Hospital, King William Street, Strand, W.C.—Clinical Assistants. Applications and testimonials to T. Beattie Campbell, Secretary, before December 1st.

Warneford Hospital, Leamington Spa.—House Surgeon; single. Salary £100 per annum, with board, lodging, and washing. Applications and testimonials to J. Warren, Secretary, before December 5th.

West London Hospital, Hammersmith Road, W.—House Physician and House Surgeon; tenable for six months. Board and lodging are provided. Applications and testimonials to R. J. Gilbert, Secretary Superintendent, before December 12th.

THE STUDENT OF MEDICINE—*continued.*

**Non-Resident Medical Officers.**

Clogher Union, Ballygawley Dispensary.—Medical Officer. Salary £75 per annum, with £15 yearly as Medical Officer of Health, together with registration and vaccination fees. Applications to Mr. John Spear Gervan, Honorary Secretary, Stewart Arms, Ballygawley. Election will take place on November 27th.

Hospital of St. Peter's Port, Guernsey.—Two qualified Surgeons (non-resident) for Hospital and Outdoor Poor. Salary £50 per annum currency (vaccination included). Applications and testimonials to N. Ferguson, President Poor-Law Board, by November 28th.

West London Hospital, Hammersmith Road, W.—Assistant Anæsthetist (Honorary and Non-Resident). Applications and testimonials to R. J. Gilbert, Secretary Superintendent, before December 12th.

WARNEFORD HOSPITAL, LEAMINGTON SPA.

HOUSE SURGEON.

